# Genetically Encoded Red Photosensitizers with Enhanced Phototoxicity

**DOI:** 10.3390/ijms21228800

**Published:** 2020-11-20

**Authors:** Dmitry A. Gorbachev, Dmitry B. Staroverov, Konstantin A. Lukyanov, Karen S. Sarkisyan

**Affiliations:** 1 Center of Life Sciences, Skolkovo Institute of Science and Technology, 121205 Moscow, Russia; igorbachev@icloud.com (D.A.G.); k.lukyanov@skoltech.ru (K.A.L.); 2 Institute of Translational Medicine, Pirogov Russian National Research Medical University, 117997 Moscow, Russia; dstaroverov1@mail.ru; 3 Shemyakin-Ovchinnikov Institute of Bioorganic Chemistry, Russian Academy of Sciences, 117997 Moscow, Russia; 4 Synthetic Biology Group, MRC London Institute of Medical Sciences, London W12 0NN, UK; 5 Institute of Clinical Sciences, Faculty of Medicine, Imperial College London, London W12 0NN, UK

**Keywords:** optogenetics, genetically encoded photosensitizers, fluorescent proteins, phototoxicity, KillerRed, SuperNova

## Abstract

Genetically encoded photosensitizers are increasingly used as optogenetic tools to control cell fate or trigger intracellular processes. A monomeric red fluorescent protein called SuperNova has been recently developed, however, it demonstrates suboptimal characteristics in most phototoxicity-based applications. Here, we applied directed evolution to this protein and identified SuperNova2, a protein with S10R substitution that results in enhanced brightness, chromophore maturation and phototoxicity in bacterial and mammalian cell cultures.

## 1. Introduction

Genetically encoded photosensitizers are proteins that induce cellular toxicity when illuminated with light. In this family of optogenetic tools, the toxicity is typically caused by reactive oxygen species generated upon absorption of photons by an aromatic moiety within the protein, called chromophore. Based on the ability to form chromophores, genetically encoded photosensitizers can be divided into two groups: those that belong to the green fluorescent protein (GFP) family and form their chromophores autocatalytically, and those that use external ubiquitous co-factors (flavins) as chromophores. Both groups are successfully used in biological studies, from ablation of cell populations [1] and inactivation of target proteins to triggering of signaling cascades with reactive oxygen species [2] or serving as tags for electron microscopy [3].

Each group has its advantages and disadvantages. For example, proteins with exogenous chromophores produce predominantly singlet oxygen and, as a result, display higher phototoxicity [4]. At the same time, being dependent on the presence of cofactors, these proteins do not always work in tumors or specific cell compartments [5]. In contrast, GFP-like phototoxic fluorescent proteins do not have these drawbacks, but generally demonstrate lower phototoxicity.

Besides phototoxicity, another practically important property of photosensitizers is their oligomeric structure. Monomeric photosensitizers are generally preferred, as they can be fused to other proteins for targeted delivery of reactive oxygen species, without forming oligomeric structures and interfering with normal cell physiology. Recently, a monomeric photosensitizer, called SuperNova [6], has been created through mutagenesis of the dimeric phototoxic red fluorescent protein KillerRed [7]. In our experiments, however, SuperNova’s phototoxicity was suboptimal for most applications, suggesting that further mutagenesis was required for optimal performance. 

Directed evolution of phototoxicity in fluorescent proteins has proved to be difficult, partly due to insufficient understanding of its structural determinants but largely due to the lack of a suitable high-throughput screening method. None of the existing phototoxic fluorescent proteins of the GFP family had its phototoxicity improved through directed evolution, either through rational design or random mutagenesis.

In our previous work on phototoxic fluorescent proteins [8], we noticed that in many cases improvements in speed and completeness of chromophore maturation were correlated with the apparent phototoxicity in vivo. This was especially evident in red fluorescent proteins that form their chromophores through a multi-stage oxidation process, with several possible final products. In red fluorescent proteins, such as KillerRed, only the final red form of the chromophore is responsible for the generation of the reactive oxygen species [9,10], explaining the observed link between the efficiency of chromophore maturation and the apparent phototoxicity. 

In this work, we aimed to apply this observation to identify mutants of the monomeric fluorescent protein SuperNova with improved folding efficiency and chromophore maturation rate, and potentially, with enhanced phototoxicity. 

## 2. Results

Based on their respective crystal structures [6,9,11], both KillerRed and SuperNova autocatalytically form DsRed-like chromophores. Generation of DsRed-like chromophores from amino acid residues requires oxidation and dehydration steps that can proceed via several alternative routes, leading to the formation of multiple chromophore forms, including forms emitting in blue, green and red parts of the spectrum [12]. A change in the amino acid environment of the chromophore can favor one of the routes, affecting the fraction of the corresponding form of the chromophore in the population of protein molecules [8].

**Figure 1:**
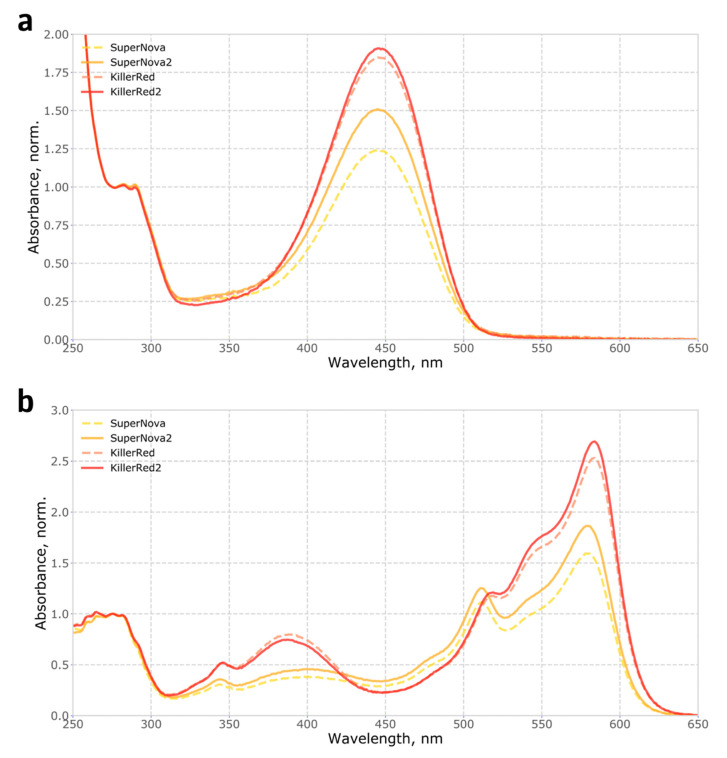
Absorbance spectra of native and alkali-denatured recombinant SuperNova, SuperNova2, KillerRed and KillerRed2 proteins: (**a**) Absorbance spectra of proteins denatured in 1 M NaOH, normalized to 280 nm; (**b**) Absorbance spectra of native proteins, in PBS, normalized to 280 nm.

To measure the completeness of chromophore maturation in the monomeric protein SuperNova and its parental dimeric protein KillerRed, we purified recombinant proteins and determined the fraction of molecules carrying any mature chromophores, in an alkaline denaturation assay which converts both the green and the red forms of the chromophore into the same spectral species (see Methods). The fraction of molecules with mature chromophores was found to be low, reaching only 35% for SuperNova and about 50% for KillerRed (Figure 1a, Methods), indicating a room for significant improvement of these proteins through directed evolution.

Therefore, we used random mutagenesis to generate a library of SuperNova mutant variants and to screen them in *E. coli*, using the brightness of colonies in the red part of the spectrum as a proxy for chromophore maturation. Among the mutants identified in this screen, one variant, dubbed SuperNova2, carried the mutation S10R and displayed significantly improved brightness. The introduction of the same mutation in KillerRed resulted in a variant KillerRed S10R, which we called KillerRed2. The mutation S10R is located on the first loop connecting the N-terminal α-helix to the first β-strand. It is not a part of the KillerRed dimerisation interface [6], and its side chain faces the solvent.

**Figure 2:**
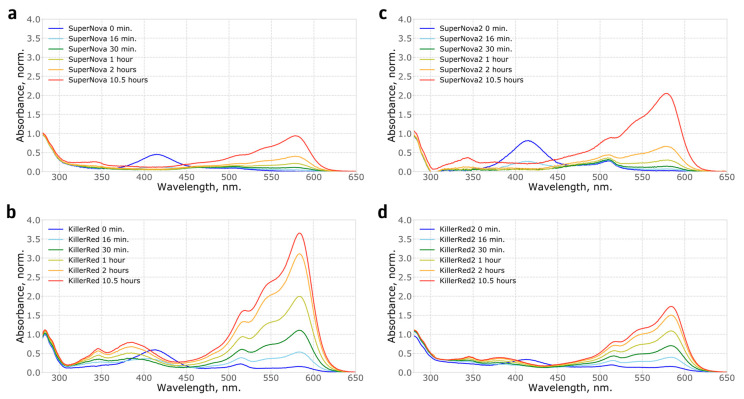
Kinetics of the absorption spectra showing chromophore maturation in vitro for recombinant: (**a**) SuperNova; (**b**) KillerRed; (**c**) SuperNova2; (**d**) KillerRed2 proteins. Spectra are normalised to the absorption at 280 nm. Notably, the completeness of chromophore maturation in these experiments was different from protein samples purified from bacterial culture likely reflecting the difference in maturation conditions of the proteins, such as temperature, pH and oxygen availability.

Mutations in loops that connect β-strands in fluorescent proteins are known to significantly affect their folding and thus the apparent brightness [13], suggesting the potential mechanism for the effect of S10R mutation. We analysed the fraction of mature chromophore and found that in SuperNova2 it has increased by 20% compared to SuperNova, while in KillerRed2 it remained largely constant, only improving by about 5% (Figure 1a). Accordingly, absorption spectra of purified proteins (Figure 1b) showed that in both mutants the S10R mutation resulted in a ~2% decrease in the content of the green form of the chromophore, and additionally, in KillerRed, in a reduction of the fraction of the 388 nm blue form. Analysis of protein maturation in vitro allowed us to determine the kinetics and relative abundance of blue, green and red forms of the chromophore (Figure 2, Supplementary Figure S1).

Notably, the fluorescence excitation and emission spectra of the mutants were similar to the parental proteins, as well as the extinction coefficient and quantum yield (Supplementary Figure S2).

**Figure 3:**
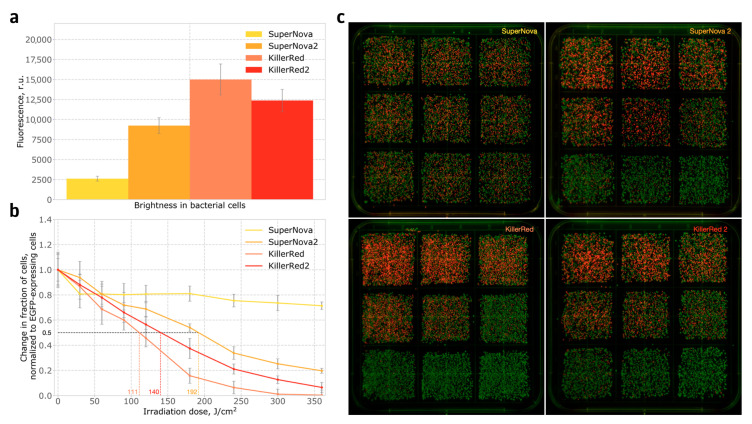
Expression of phototoxic fluorescent proteins in bacteria: (**a**) Brightness in bacterial cells after incubation for 20 h at 37 °C and another 24 h at room temperature; (**b**) Phototoxicity of SuperNova, SuperNova2, KillerRed and KillerRed2 in bacteria; (**c**) Photographs of plates with bacterial colonies expressing EGFP and one of the photosensitizers, plated after light irradiation experiment. Each squared section represents a single time point (0.0, 0.5, 1.0, 1.5, 2.0, 3.0, 4.0, 5.0 and 6.0 min, which is equivalent to the irradiation dose 0, 30, 60, 90, 120, 180, 240, 300 and 360 J/cm^2^. Ordered from left to right and from top to bottom). Indicative irradiation dose values required for ablation of 50% of cells are shown on the X axis in colour.

We then tested how S10R mutation affected the in-cell apparent brightness and phototoxicity. In bacteria, the brightness of SuperNova2-expressing colonies was found to be over four-fold higher than that of SuperNova-expressing colonies. At the same time, KillerRed2 was slightly dimmer than the parental protein (Figure 3a).

The phototoxicity in bacteria was measured by assessing the changes in the ratio of red to green cells in mixtures of bacteria expressing phototoxic proteins and bacteria expressing EGFP. The experiments were performed after 20 h incubation at 37 °C and subsequent 24 h incubation at room temperature. Briefly, we mixed *E. coli* cells expressing GFP and *E. coli* expressing a phototoxic protein in 1:1 ratio, and the population was subjected to light irradiation for a variable amount of time. The cells were then plated and, on the next day, green and red colonies were counted, and phototoxicity was calculated from the change in ratio of red to green colonies (see Methods). At all time points, SuperNova2 demonstrated significantly higher phototoxicity than SuperNova, with almost 4-fold difference in the amount of dead cells observed after six minutes of irradiation (Figure 3b,c), while the phototoxicity of KillerRed2 was slightly lower than that of KillerRed. Interestingly, when the experiment was performed without a 24-h incubation at room temperature, the phototoxicity of SuperNova was negligible, the phototoxicity of KillerRed and KillerRed2 was similar, and the phototoxicity of SuperNova2 was close to that of KillerRed (Supplementary Figure S3). This indicates that S10R mutation improves protein maturation at 37 °C. 

**Figure 4:**
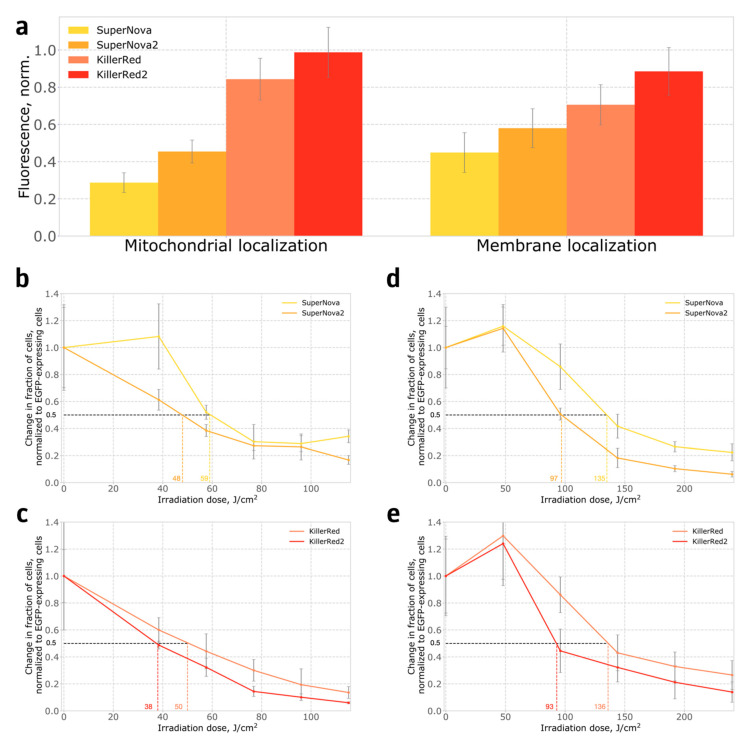
Expression of phototoxic fluorescent proteins in HeLa Kyoto cells: (**a**) Brightness of cells expressing phototoxic proteins targeted to mitochondria, or anchored in the cell membrane; (**b**,**c**) Phototoxicity of proteins targeted to mitochondria; (**d**,**e**) Phototoxicity of proteins anchored on the cell membrane. Indicative irradiation dose values required for ablation of 50% of cells are shown on the X axis in colour.

We also compared the phototoxicity of SuperNova, SuperNova2, KillerRed and KillerRed2 in stably transformed HeLa Kyoto human cell lines, where proteins were either targeted to mitochondria, or anchored in the plasma membrane. In contrast to bacteria, in the mammalian system both SuperNova2 and KillerRed2 were brighter than their parental proteins (Figure 4a), and also demonstrated higher phototoxicity (Figure 4b–e). The S10R mutation likely leads to a more complete maturation of the chromophore, which is evident from the increase in the apparent in-cell brightness. We believe that the increase in the fraction of the protein with mature chromophore leads to the increase in the formation of reactive oxygen species and, consequently, phototoxicity. Interestingly, in cells expressing photosensitizers targeted to the cell membrane, low light doses increased cell proliferation, in accordance with observations described in the literature [14,15].

**Figure 5:**
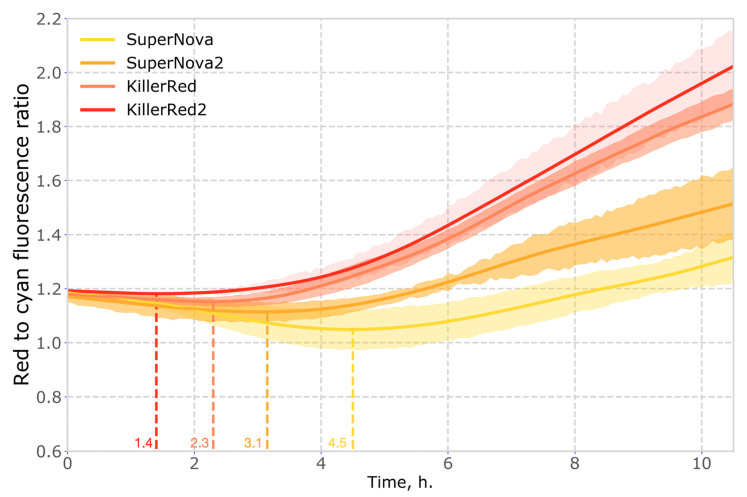
Time evolution of red to cyan fluorescence ratio in mammalian cells coexpressing red phototoxic proteins and mTurquoise2. Time corresponding to the extremum of the curve (when the rate of accumulation of red fluorescence exceeds that of cyan fluorescence) is shown on the X axis in colour.

Another important property of phototoxic fluorescent proteins is their chromophore maturation rate. Faster maturation results in higher concentration of phototoxic molecules, which translates non-linearly into the observed level of phototoxicity. To compare the maturation rate of the red genetically encoded photosensitizers, we used co-expression with cyan fluorescent protein mTurquoise2 as a reference. We found that in mammalian cells the maturation rates of KillerRed2 and SuperNova2 were higher than those of parental proteins (Figure 5, Supplementary Figure S5).

## 3. Discussion and Conclusions

To conclude, here we report two variants of phototoxic red fluorescent proteins KillerRed and SuperNova, carrying the substitution S10R. This mutation significantly improved brightness, maturation rate and phototoxicity in the genetic background of the parental protein SuperNova in all tested experimental systems. In the background of KillerRed, the S10R mutation effect was less definite than in the background of SuperNova, leading to slightly lower phototoxicity in bacteria but higher phototoxicity and brightness in mammalian cells. 

We believe that the effect of the S10R mutation in KillerRed2 is temperature dependent. At 37 °C KillerRed2 is comparable in phototoxicity to KillerRed in bacteria (Supplementary Figure S3) and exceeds its phototoxicity in mammalian cells (Figure 4c,e). However, the maturation efficiency of KillerRed2 at room temperature is almost two times lower than that of KillerRed (Figure 2b,d), which in turn affects its phototoxicity (Figure 3b, Section 4). Interestingly, we did not observe this effect in SuperNova2, which has greater maturation efficiency and phototoxicity than SuperNova, both at 37 °C and at room temperature. 

In all experiments, the proposed link between protein brightness and phototoxicity was confirmed, suggesting that in-cell apparent brightness can be used as a proxy for phototoxicity in directed evolution of photosensitizers with incomplete chromophore maturation. While SuperNova2 outperforms SuperNova in all conditions tested in this study, its photototoxicity is generally lower than the phototoxicity of KillerRed and KillerRed2 (perhaps, due to lower photostability, Supplementary Figure S4). Thus, we recommend using KillerRed2 with mitochondrial localisation for applications requiring light-induced cell death, while for targeted inactivation of cellular proteins we recommend using SuperNova2 to avoid unintended dimerisation-induced physiological effects. 

Phototoxic proteins SuperNova2 and KillerRed2 reported here will enable phototoxicity experiments with lower light doses and shorter exposure times, and can serve as a useful template for further mutagenesis to generate new genetically encoded photosensitizers.

## 4. Materials and Methods 

### 4.1. Plasmid Construction and Mutagenesis 

Plasmids were constructed by Golden Gate assembly, following the MoClo syntax as described in [16]. SuperNova and KillerRed sequences were domesticated by removal of BsaI and BpiI restriction sites, and ordered as synthetic DNA. For bacterial expression, the genes were cloned into a MoClo Level 1-like vector under the control of araBAD promoter. Random mutagenesis was performed using a GeneMorph II Random Mutagenesis Kit. Cloning of the mutant library was also carried out by the Golden Gate assembly using BsaI restriction sites. For expression in eukaryotic cells and creating stable cell lines, we used a domesticated pLVT vector, with BsaI and BpiI recognition sites removed [17]. Sequences of all vectors used in this study available as Supplementary Table S1.

### 4.2. Purification of Recombinant Proteins

To purify recombinant proteins, we inoculated 200 μL of an overnight culture of *E. coli* TOP10 cells expressing a fluorescent protein gene into a 800 mL flask containing 200 mL of LB medium with ampicillin (100 mg/mL). The flask was then placed in a thermostated shaker for 3 h (37 °C, 250 rpm). After 3 h, a transcription inducer, arabinose, was added to the cell culture to a final concentration of 10 mM, and the flask was incubated for another 20 h. 

All following operations were carried out on ice. The cell culture was centrifuged in a Heraeus Multifuge centrifuge (Thermo Fisher Scientific, Waltham, MA, USA) for 20 min at 4500 rpm at 4 °C. The supernatant was discarded, and the pellet was resuspended in 4 mL of phosphate-buffered saline (PBS, pH 7.4). The cells were disrupted by ultrasound on a Vibra Cell sonicator (Sonics Newtown, CT, USA) with the following program: 5 s—on cycle, 10 s—off-cycle, 40 cycles; amplitude—30%. The cell suspension was then centrifuged in a 5415R centrifuge (Eppendorf, Hamburg, Germany) at 13,000 rpm at 4 °C. The supernatant was transferred into a clean tube with 400 μL of Talon metal affinity resin (Takara Bio, Mountain View, CA, USA) equilibrated with PBS. The tube was placed in a shaker for 1 h (200 rpm, room temperature). Thereafter, the metal-affinity resin was washed several times with PBS, and then the protein was eluted with a PBS containing 200 mM EDTA. Finally, the buffer was replaced with PBS on Amicon Ultra 0.5 mL Centrifugal Filters (Merck, Darmstadt, Germany). 

### 4.3. Characterization Proteins In Vitro 

The absorption spectra were measured on a Cary 100 BioUV-VIS spectrophotometer (Agilent, Santa Clara, CA, USA). A Cary Eclipse fluorescence spectrophotometer (Agilent) was used to measure the excitation and fluorescence spectra. The spectra of proteins were measured in phosphate-buffered saline (PBS, pH 7.4).

To accurately determine the concentration of the protein with mature chromophore, we determined the total chromophore concentration by alkali denaturation. In 0.1 N NaOH, green and red forms of DsRed-like chromophore are both converted to a species that absorbs at 450 nm, with an extinction coefficient of ∼ 44,000 M^−1^ cm^−1^ [18], allowing to determine the fraction of molecules with complete chromophore maturation by normalising to the the total protein determined by absorbance at 280 nm in 0.1 N NaOH. Extinction coefficient at 280 nm was calculated in ProtParam tool (ExPASy, SIB Bioinformatics Resource Portal, https://web.expasy.org/protparam/). 

### 4.4. Phototoxicity Assay in E. coli

*E. coli* TOP10 cells were transformed with expression vectors, where EGFP, SuperNova, SuperNova2, KillerRed or KillerRed2 genes were placed under the control of araBAD promoter. On the next day, one colony from each plate was taken and diluted in PBS. A small portion of the cell suspension containing about 5000 cells was seeded on a Petri dish and incubated for 20 h at 37 °C. The dish was then left at room temperature for 24 h to allow for better chromophore maturation. 

After that, the colonies were washed from Petri dishes and resuspended in PBS. The cell suspension was diluted several times to a concentration of 1000 cells per 100 μL. Cells with EGFP (a non-phototoxic reference protein) were mixed with cells expressing the photosensitizers in a 1:1 ratio, in the total volume of 5 mL. Before irradiation 50 µL of an unirradiated sample was spread on a Petri dish. Then, the cell suspension was irradiated and 50 µL aliquots were taken after 0.5, 1.0, 1.5, 2.0, 3.0, 4.0, 5.0, 6.0 min.

The irradiated aliquots were then plated on a Petri dish and incubated overnight at 37 °C. On the next day, green and red colonies were counted, and the ratio of red to green colonies was determined and normalised to the ratio obtained for the non-irradiated sample (0 min). Each phototoxicity experiment was carried out in four independent replicates. The samples were irradiated by five PC Amber (591 nm) Rebel LEDs (LXM2-PL01-0000, Luxeonstar, Lethbridge, AB, Canada), forward current 350 mA, 550 lm. The spectra of the LEDs are shown on the Supplementary Figure S6, and a photo of the LED irradiation setup is shown as Supplementary Figure S7.

### 4.5. Phototoxicity Assay in Mammalian Cells 

HeLa Kyoto and HEK293T cell lines were obtained from established frozen stocks of the laboratory. All mammalian cell lines were cultured in Dulbecco’s Modified Essential Medium (DMEM) containing 2 mM glutamine and 4.5 g/L glucose (PanEco, Moscow, Russia), supplemented with HyClone 10% fetal bovine serum (Thermo Fisher Scientific, Waltham, MA, USA), at 37 °C and 5% CO_2_.

When determining phototoxicity in mammalian cells, we used a ratiometric approach similar to the one used in experiments in bacteria. Nine HeLa Kyoto stable cell lines were created, expressing transgenes with one of the following structures: *CMV_promoter - mitochondrial_localization - photosensitizer - T2A - TagBFP2*, *CAG_promoter - membrane_localization - photosensitizer - T2A - TagBFP2*, *CMV_promoter - EGFP - nuclear_localization_signal*, where photosensitizer stands for SuperNova, SuperNova2, KillerRed or KillerRed2 genes. 

After transduction, cells demonstrating similar brightness in the blue channel were sorted and plated. One week after sorting, the obtained stable lines were analyzed in order to ensure that the resulting lines have a comparable level of expression, determined as the brightness in the blue channel (Supplementary Figure S8). Cell line expressing photosensitizer was then mixed with cells expressing EGFP in a 1:1 ratio, and plated into a 24 well plate, 50 thousand cells per well. The next day, the medium was changed to PBS. The plate was placed between two LED assemblies in such a way that one well was irradiated with two diodes from the top and the bottom (Supplementary Figure S9). Each well was irradiated by two orange LEDs 592 nm, forward current 500 mA, 160 mW/cm^2^. The spectra of the LED are shown on the Supplementary Figure S6. There were six time points in the experiments (0, 4, 6, 8, 10, 12 min for mitochondrial localization and 0, 5, 10, 15, 20, 25 min for membrane localization), with four wells measured for each time point. 

After irradiation, the PBS solution was replaced with the full DMEM media, and plates were placed in an incubator for 24 h. In each well, an average of 300–400 cells were photographed in one field of view in a Leica DMI6000b inverted microscope (Leica Microsystems, Wetzlar, Germany) equipped with a HCX PL FLUOTAR L 20×/0.40 lens. The number of cells expressing EGFP or photosensitizer was counted using ImageJ software. For each time point, the ratio of red and green cells was determined, and normalized to the ratio in the non-irradiated sample (time point 0). For the detection of green fluorescence, GFP filter cube was used (excitation filter 470/40 nm emission 525/50 nm, 495 nm dichroic mirror, Leica), for the red fluorescence–mCherry/TFT filter cube (excitation filter 578/21 nm, emission filter 641/75 nm, 596 nm dichroic mirror, Semrock, Rochester, NY, USA).

### 4.6. Measurements of Chromophore Maturation Rates In Vivo 

The protocol for determining the chromophore maturation rate in vivo was based on the protocol given in the reference [19]. HEK293T cells were seeded in 8-well µ-Slide (80826, Ibidi, Gräfelfing, Germany). On the next day, cells were transfected with 300 ng of a pCMV-mTurquoise2-T2A_peptide-photosensitizer plasmid, and 1 μg PEI in 200 μL Opti-MEM. Four hours after transfection, Opti-MEM was changed to the imaging media (MEM media without sodium bicarbonate with the addition of 20 mM HEPES) and a 8-well slide was placed on the microscope. In this experiment, the sample was kept at 37 °C. For the detection of cyan fluorescence, CFP filter cube was used (excitation filter 448/20 nm emission 482/25 nm, 466 nm dichroic mirror, Semrock, Rochester, NY, USA), for the red fluorescence–mCherry/TFT filter cube (excitation filter 578/21 nm, emission filter 641/75 nm, 596 nm dichroic mirror, Semrock, Rochester, NY, USA). Images in each of these fluorescence channels were acquired every 5 min for the total duration of 24 h. 

Using ImageJ software tools, a region of interest (ROI) was drawn around each individual cell and the fluorescence values were corrected for the background fluorescence. The mean fluorescence in each frame was then extracted by the Z-stack profile function and all resulting curves were synchronized to the starting time point. 

Since the increase in fluorescence in a function of not only the kinetics of maturation but also the kinetics of protein synthesis, we normalized the fluorescence in the red channel to the fluorescence in the cyan channel to obtain the relative kinetics of maturation of red proteins. The curves obtained for each cell were averaged and the standard deviation was found. The number of cells analysed in these experiments was: *N* = 15 for SuperNova, *N* = 12 for SuperNova2, *N* = 20 for KillerRed, and *N* = 14 for KillerRed2.

### 4.7. Measurements of Chromophore Maturation Rates In Vitro

Measurements of chromophore maturation rates require anaerobic conditions. We expressed phototoxic proteins in *E. coli*, using degassed LB medium. 200 μL of an overnight culture of *E. coli* TOP10 cells was inoculated into 200 mL of the degassed LB medium with ampicillin (100 mg/mL) in a 500 mL sealed flask. The flask was then placed in a thermostated shaker for 3 h (37 °C, 250 rpm). After 3 h, a degassed arabinose solution was added to the culture to a final concentration of 10 mM, and the flask was put back into the shaker for another 20 h. 

After that, the sealed flask was transferred to an ice water bath for 30 min. All subsequent manipulations were carried out very quickly and took place on ice. The cell culture was centrifuged for 10 min at 4500 rpm at 4 °C. The supernatant was discarded, and the pellet was resuspended in 6 mL degassed PBS, pH 7.4. The cells were disrupted by ultrasound using a Sonics Vibra Cell sonicator (program: 10 s—on cycle, 5 s—off-cycle, 40 cycles; amplitude—30%). The cell suspension was then centrifuged in an Eppendorf 5415R centrifuge at 13,000 rpm at 4 °C for 10 min. The supernatant was transferred to a 5 mL Eppendorf with 200 μL of Talon metal affinity resin (Takara Bio, Mountain View, CA, USA) equilibrated with degassed PBS. The tube was shaken in an ice water bath for 10 min. Thereafter, the metal-affinity resin with protein was washed three times with 5 mL degassed PBS. The protein was then eluted with a 200 μL PBS containing 200 mM EDTA. 

The concentrated protein solution was divided into two parts. One part was diluted 10 times and placed in a cuvette for measuring absorption spectra kinetics, the other was diluted 50 times and placed in a cuvette for measuring fluorescence kinetics. To visualise the maturation of various forms of the chromophore, we normalized the absorption spectra to 280 nm. 

### 4.8. Measurements of Fluorescence Protein Photostability

Photobleaching was carried out using a wide-field Leica DMI6000b inverted microscope (Leica Microsystems) equipped with HC PL Apo 40 × 0.85 lens, which made it possible to visualize about 200 cells in one field of view. We used HeLa Kyoto stable cell line, expressing KillerRed, SuperNova and their mutants with mitochondrial or membrane localisation. For the photobleaching acquisition we used mCherry/TFT filter cube (excitation filter 578/21 nm, emission filter 641/75 nm, 596 nm dichroic mirror, Semrock). The cells were continuously irradiated with light passing through the excitation filter for 10 min. Light intensity above the objective at the focal plane corresponded to 10 W/cm^2^. During photobleaching, the camera acquired images every 10 s. 

Using ImageJ software tools, a region of interest (ROI) was drawn around each individual cell and the fluorescence values were corrected for the background fluorescence. The mean fluorescence in each frame was then extracted and normalized to the first frame for plotting photobleaching curves.

## Supplementary Materials

Supplementary materials can be found at https://www.mdpi.com/1422-0067/21/22/8800/s1.
