# Supplementary material for: Genetically Encoded Red Photosensitizers with Enhanced Phototoxicity
Source: Int J Mol Sci. 2020 Nov 20;21(22):8800. doi: 10.3390/ijms21228800 (PMC7709005; doi:10.3390/ijms21228800)
Supplement: Supplementary file 1 [file ijms-21-08800-s001.zip › Supplementary/ijms-979369-supplementary-2.docx]

**Supplementary Figures**


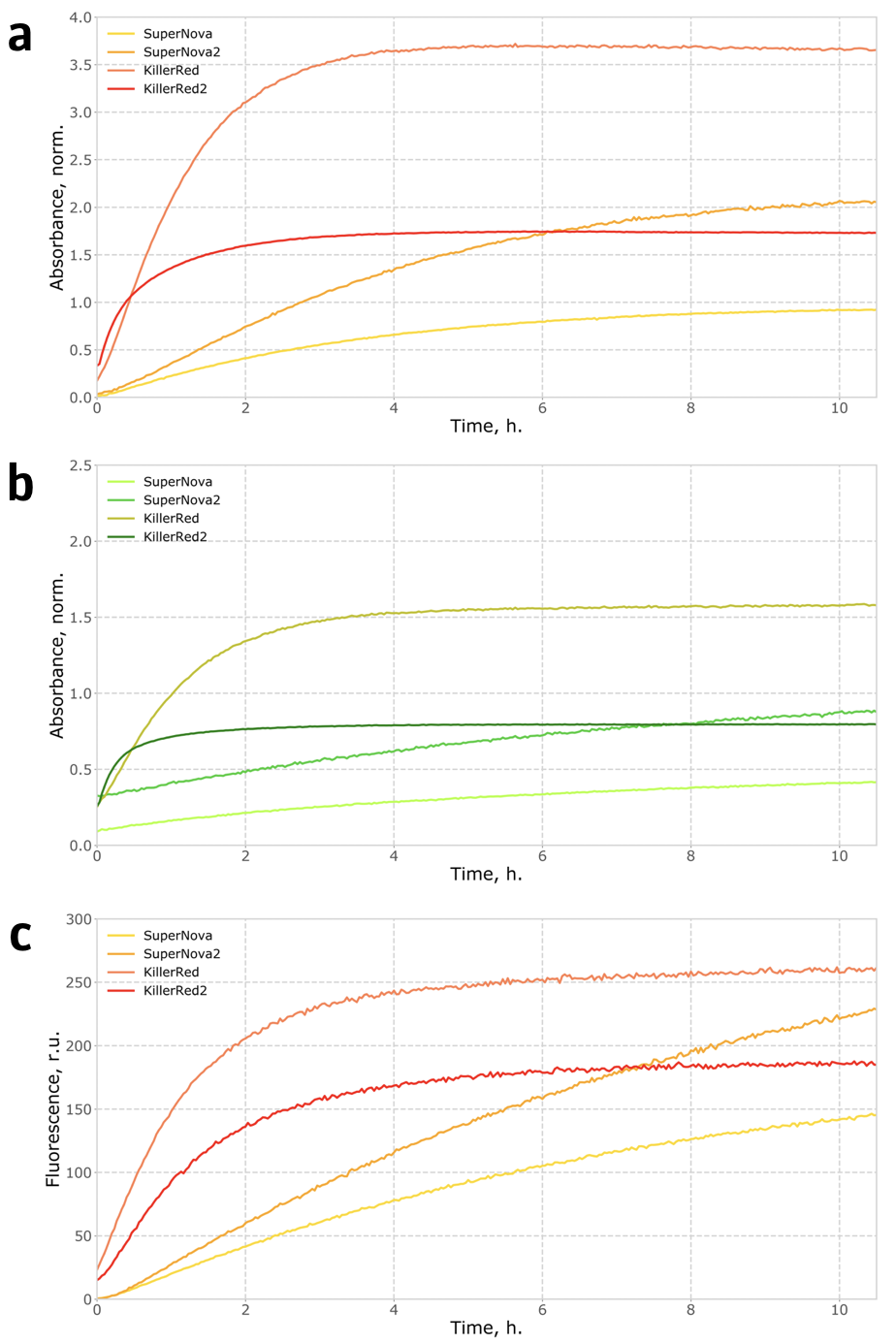


**Supplementary Figure S1**. Kinetics of chromophore maturation of recombinant SuperNova, SuperNova2, KillerRed and KillerRed2 *in vitro*. (**a**). Kinetics of absorbance spectra of the red form of the chromophore (579 nm for SuperNova, SuperNova2 and 584 nm for KillerRed, KillerRed2). Absorbance spectra were normalised to the absorption at 280 nm. (b). Kinetics of absorbance spectra of the green form of the chromophore (510 nm for SuperNova, SuperNova2 and 515 nm for KillerRed, KillerRed2). Absorbance spectra were normalised to the absorption at 280 nm. (**c**). Kinetics of emission spectra of the red form of the chromophore (607 nm for SuperNova, SuperNova2 and 615 nm for KillerRed, KillerRed2).


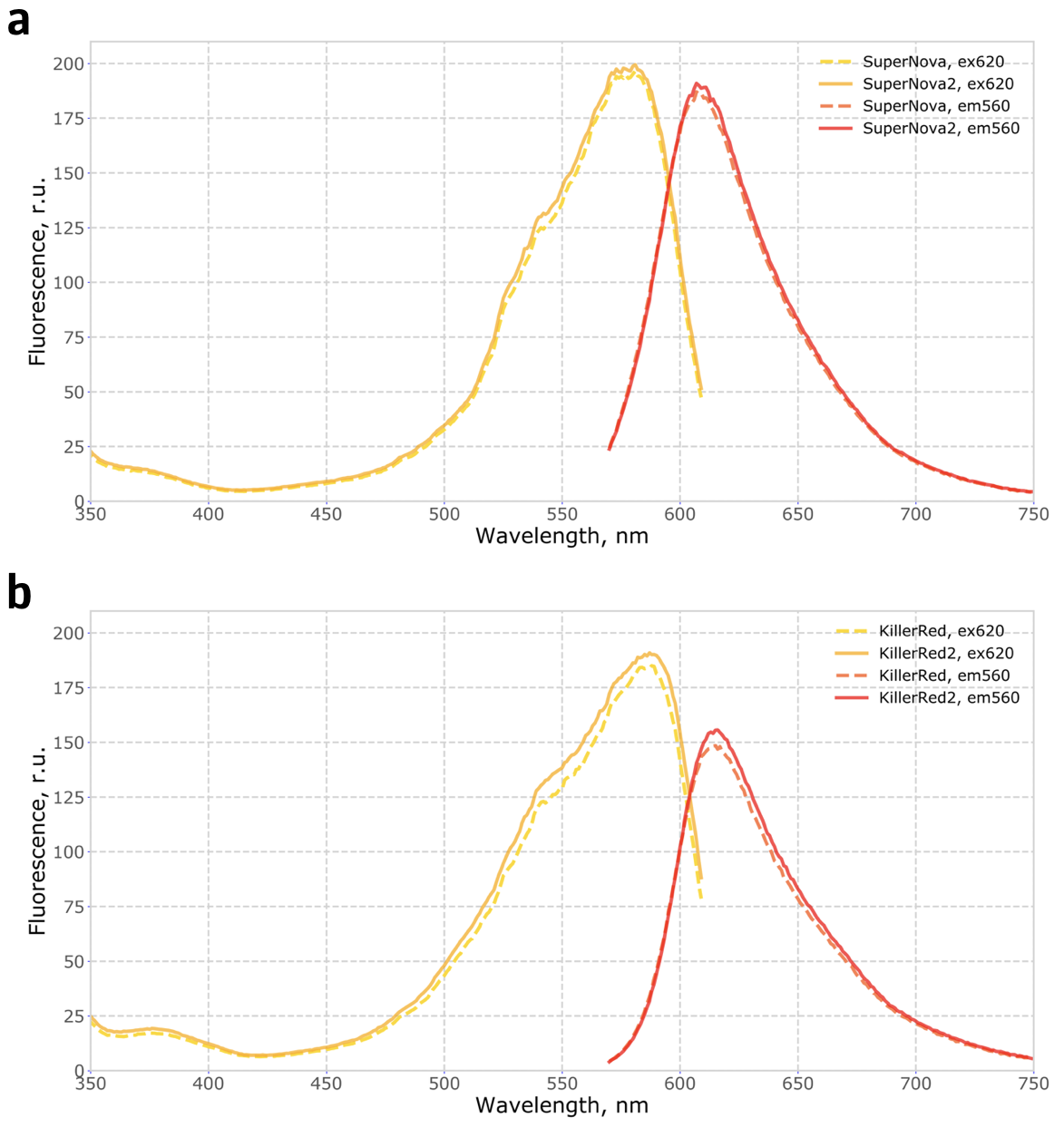


**Supplementary Figure S2**. Fluorescence excitation spectra (emission registered at 620 nm) and emission spectra (with excitation at 560 nm) of purified recombinant SuperNova, SuperNova2 (a), KillerRed and KillerRed2 (b). Protein concentrations in the samples were adjusted to result in optical density of OD=0.05 at the corresponding absorption maximum (absmax580 nm for SuperNova, SuperNova2; absmax 590nm for KillerRed, KillerRed2). Thus, the similarity of the emission spectra suggests similar values for fluorescence quantum yield between the parental proteins and their mutants.


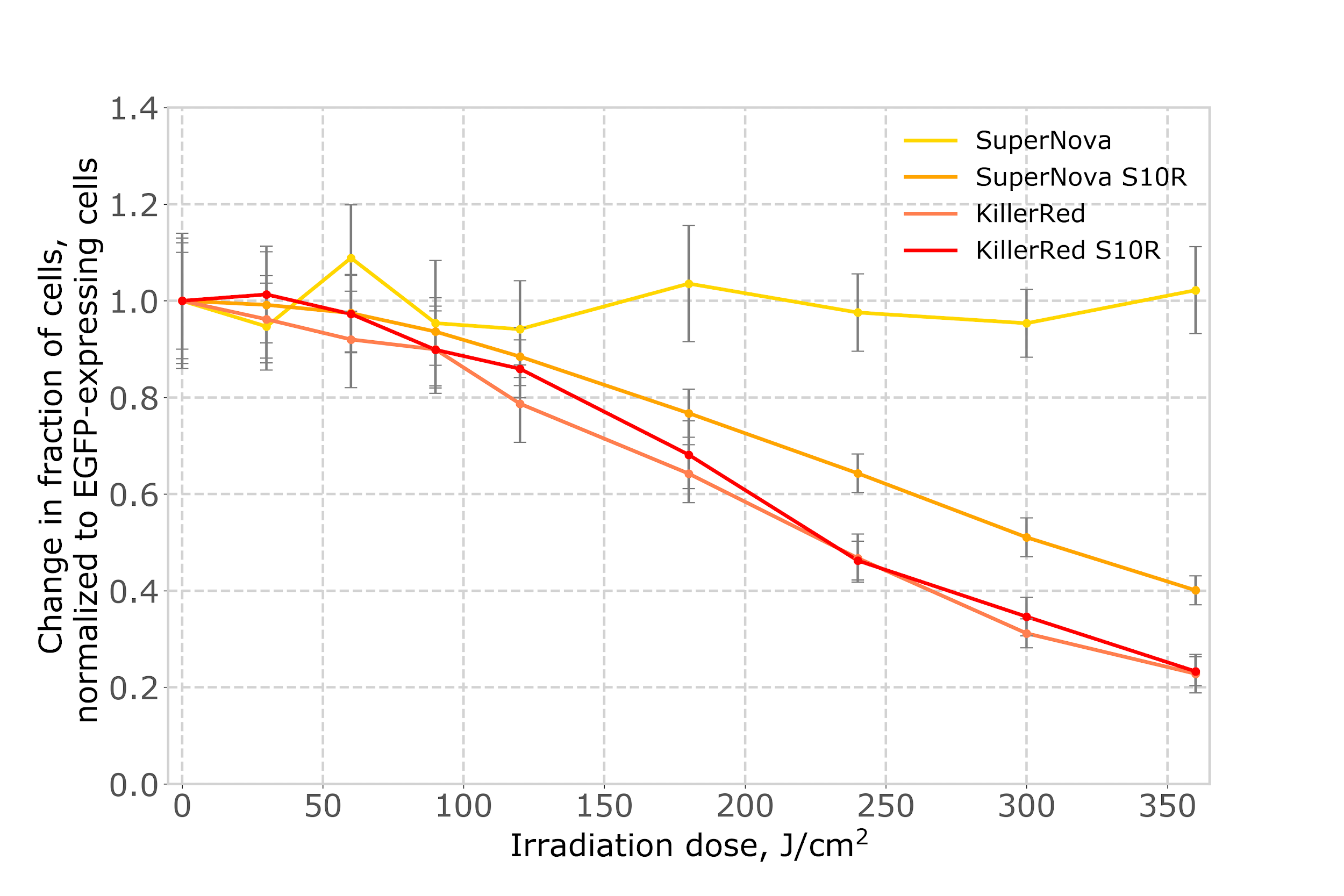


**Supplementary Figure S3.** Phototoxicity of SuperNova, SuperNova2, KillerRed and KillerRed2 in bacteria (experiment performed after 20-hour incubation at 37°C, without additional incubation at room temperature).


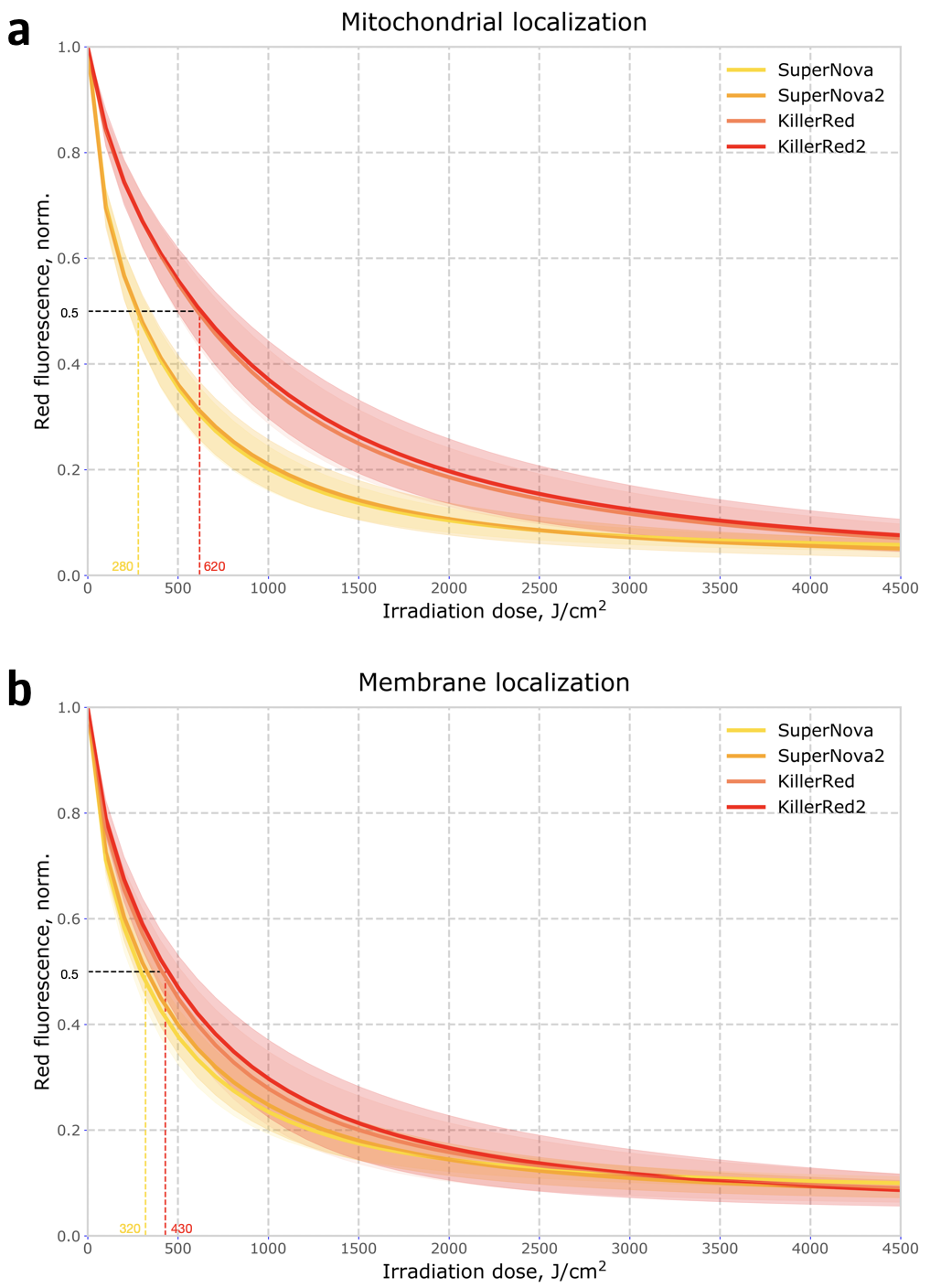


**Supplementary Figure S4.** Fluorescent proteins photostability *in vivo*. Irradiation dose required for half-bleaching of corresponding cells is indicated on X-axis in colour.


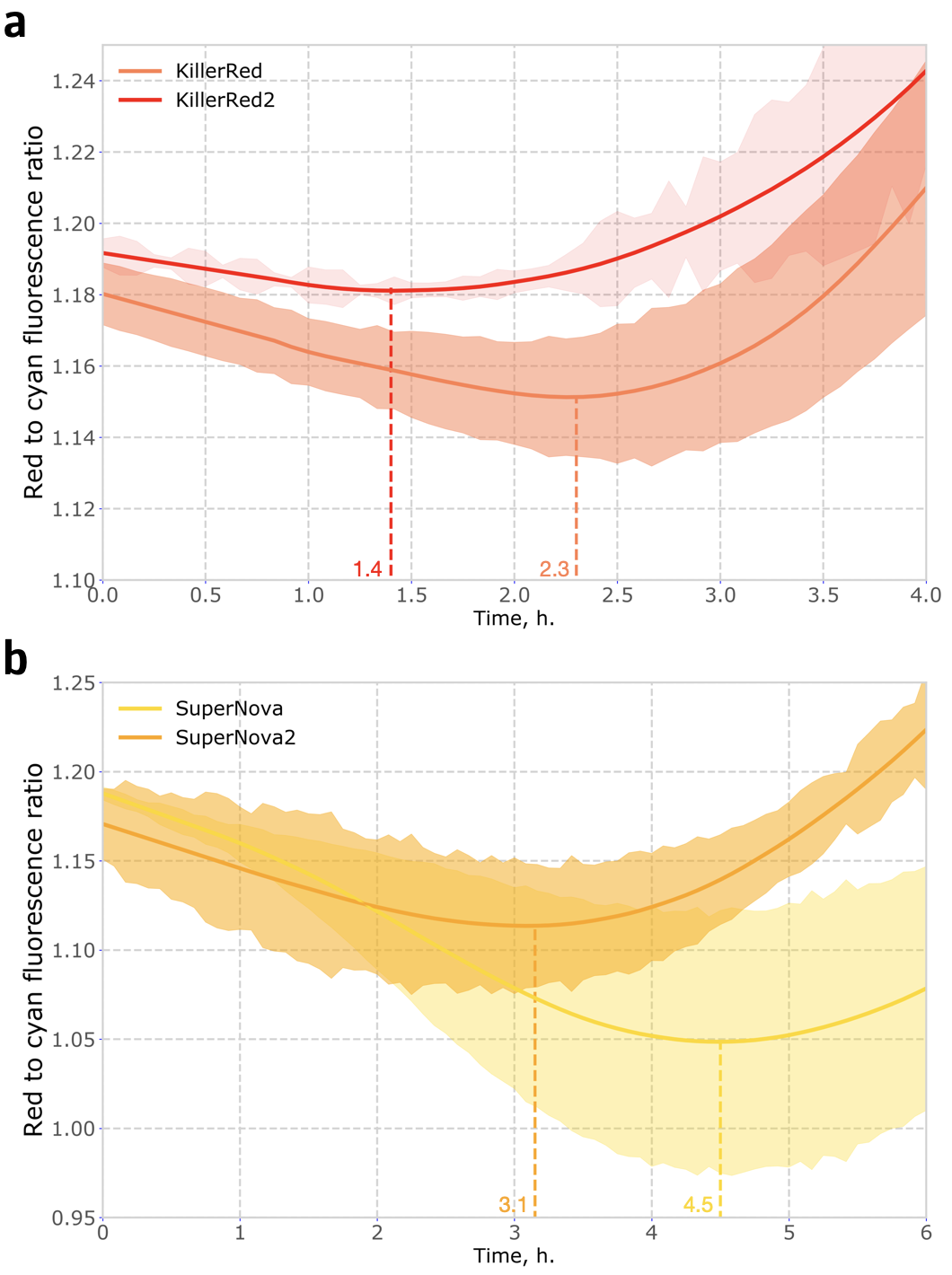


**Supplementary Figure S5**. Time evolution of red to cyan fluorescence ratio in mammalian cells coexpressing red phototoxic proteins and mTurquoise2. KillerRed, KillerRed2 (a) and SuperNova, SuperNova2 (b). Time corresponding to the extremum of the curve (when the rate of accumulation of red fluorescence exceeds that of cyan fluorescence) is shown on the X axis in colour.


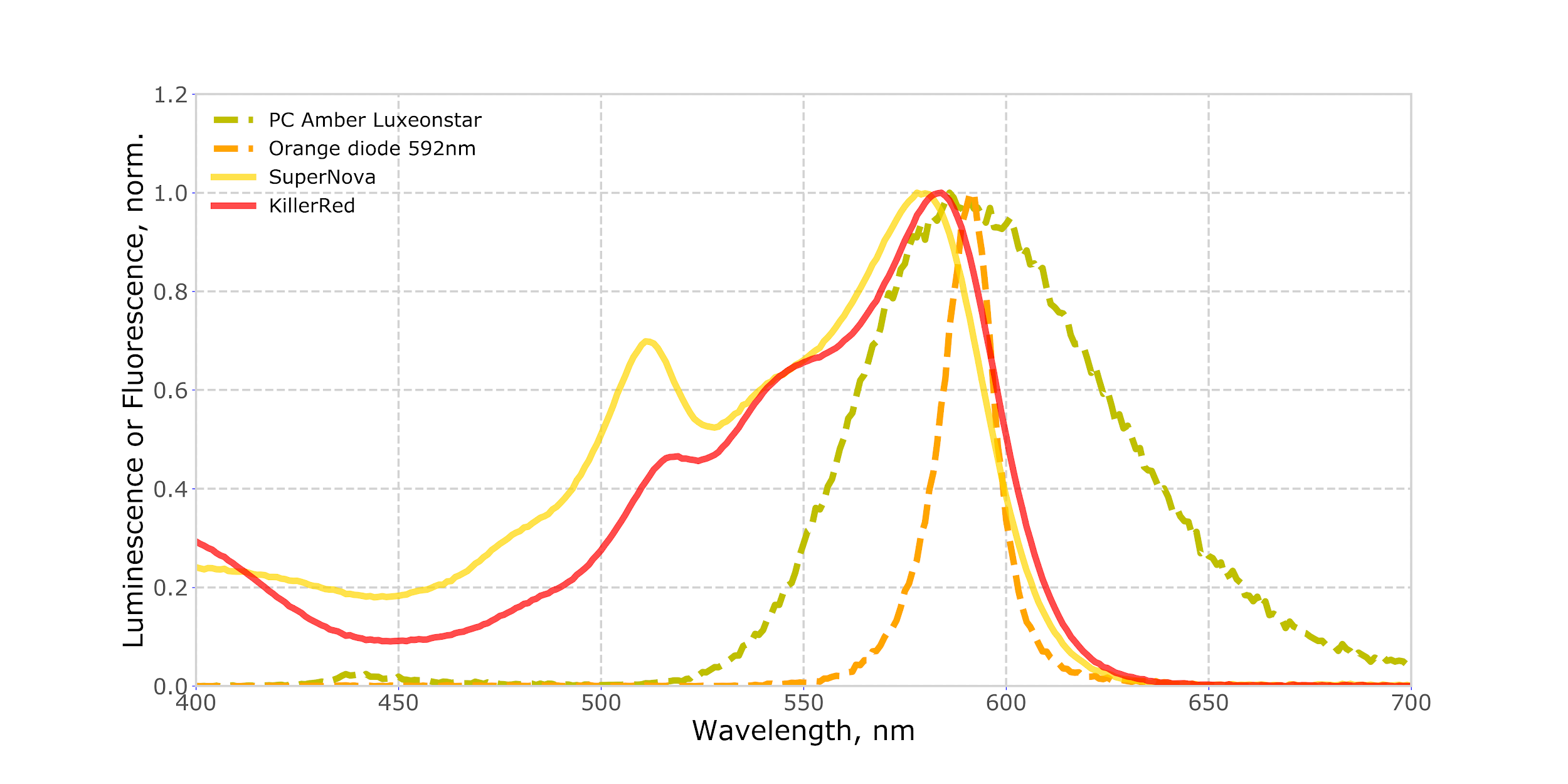


**Supplementary Figure S6**. Overlay of LED emission spectra and KillerRed and SuperNova absorbance spectra.


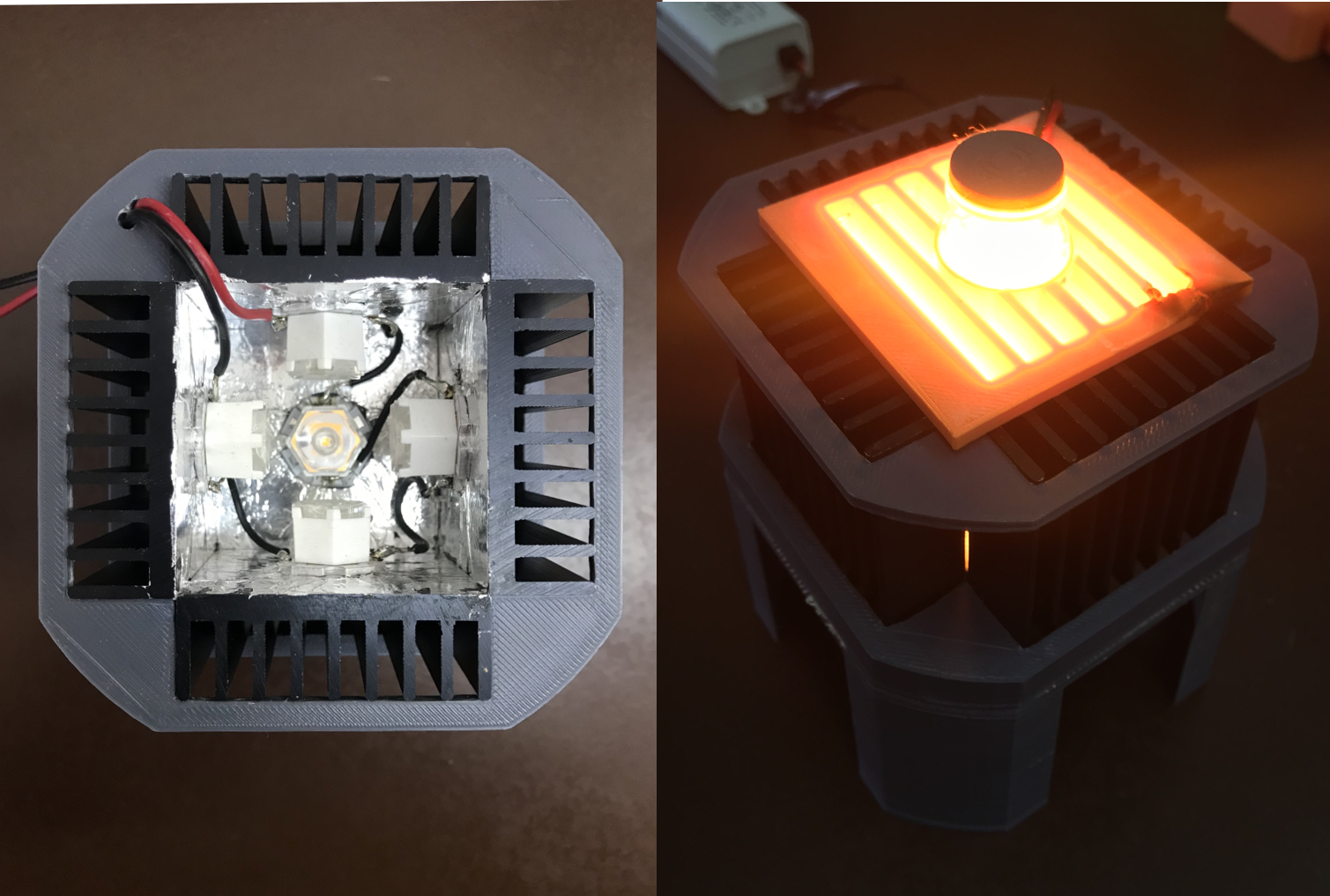


**Supplementary Figure S7.** Photographs of LED irradiation setup used in phototoxicity assay in *E. coli. Irradiation power used in the experiments was 1 W/cm2.*


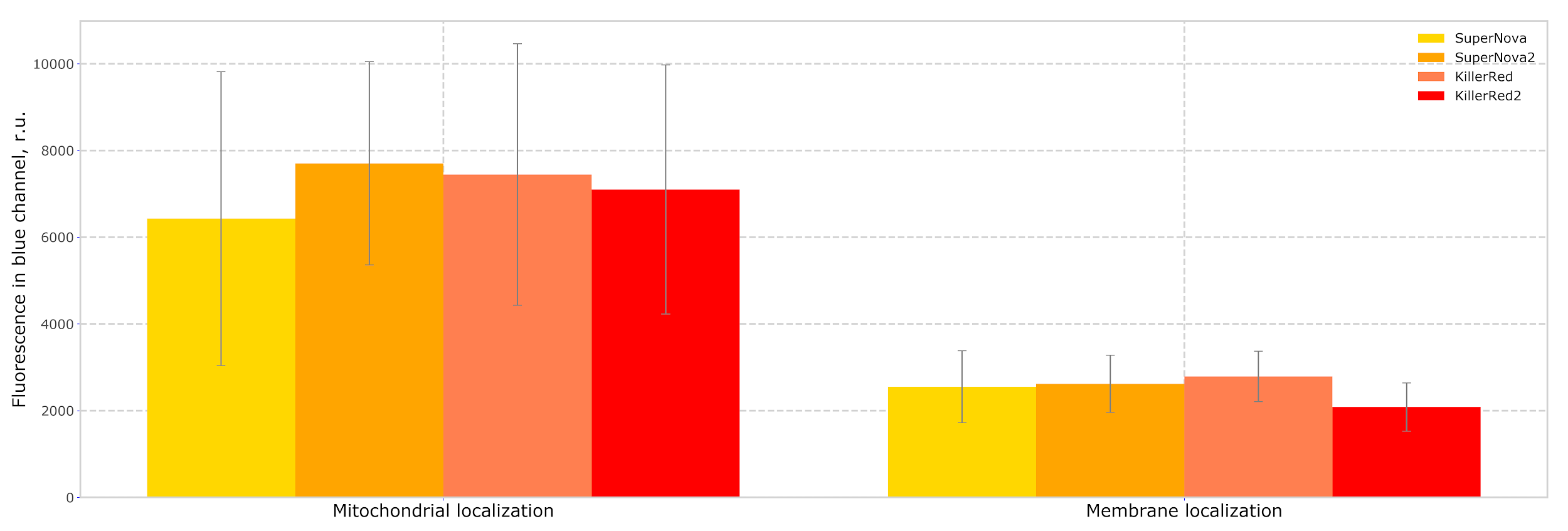


**Supplementary Figure S8.** Brightness of blue fluorescence of cell lines stably expressing SuperNova-2A-TagBFP2, SuperNova2-2A-TagBFP2, KillerRed-2A-TagBFP2, and KillerRed2-2A-TagBFP2 genes.


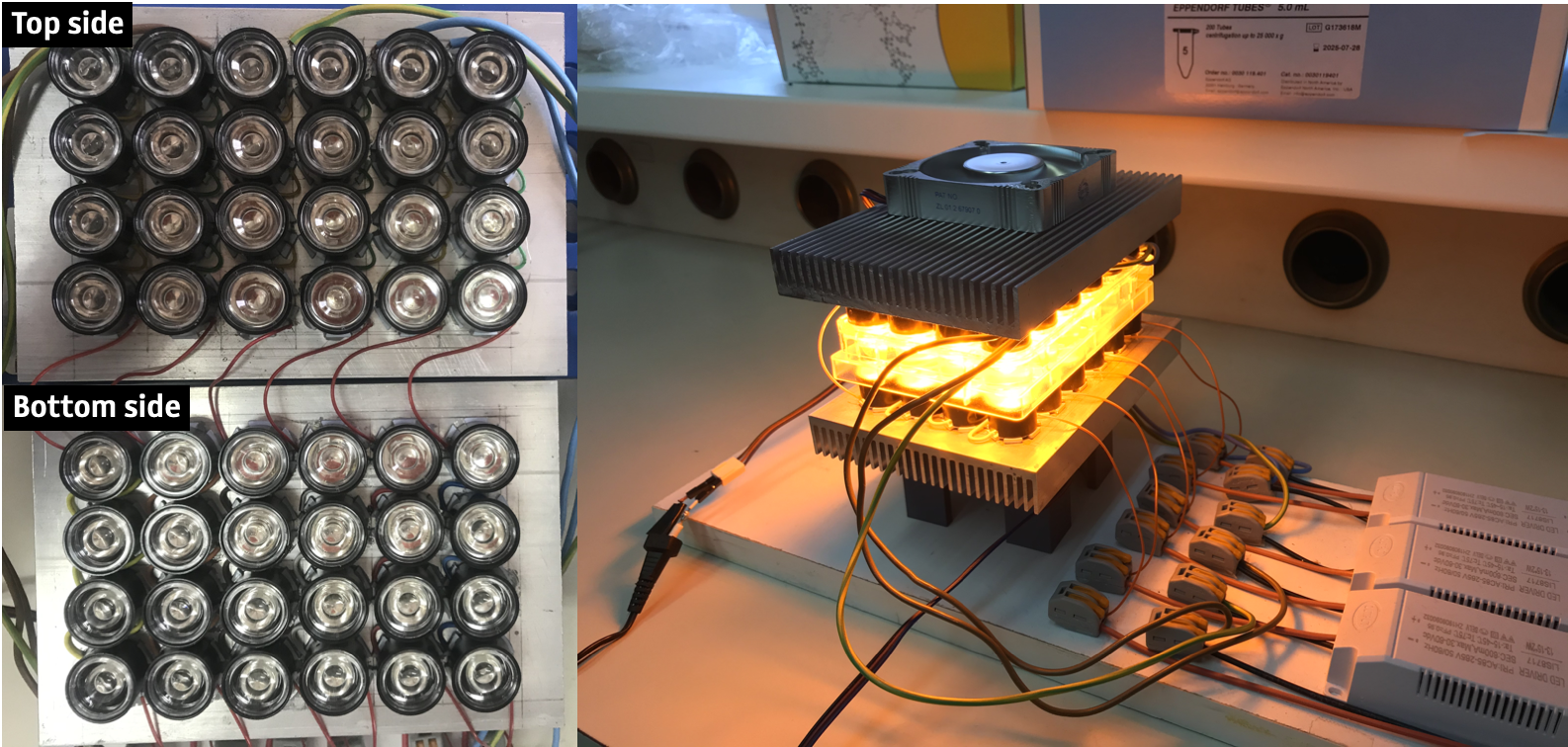


**Supplementary Figure S9.** Photographs of LED irradiation setup used in phototoxicity assay in mammalian cells. Irradiation power used in the experiments was 160 mW/cm^2^ per well.

**Table S1**. DNA sequences of the constructs used in this study.

| CMV_promoter-mitochondrial_localization-KillerRed-T2A-TagBFP2 | <https://benchling.com/s/seq-q81hU8i5nxrNxS9xg1KK> |
| --- | --- |
| CMV_promoter-mitochondrial_localization-KillerRed2-T2A-TagBFP2 | <https://benchling.com/s/seq-2UH2fO3ixtrSGHZHNNzk> |
| CMV_promoter-mitochondrial_localization-SuperNova-T2A-TagBFP2 | <https://benchling.com/s/seq-Eav8XMTyfUhm9nhuWehN> |
| CMV_promoter-mitochondrial_localization-SuperNova2-T2A-TagBFP2 | <https://benchling.com/s/seq-qPfyQlaoWpboVhlaRFuF> |
| CAG_promoter-membrane_localization-KillerRed-T2A-TagBFP2 | <https://benchling.com/s/seq-7PlMGT1oR0khAcMpH5AL> |
| CAG_promoter-membrane_localization-KillerRed2-T2A-TagBFP2 | [https://benchling.com/s/seq-pzv7lIU9vHd9yMjaExlM](https://benchling.com/s/seq-7PlMGT1oR0khAcMpH5AL) |
| CAG_promoter-membrane_localization-SuperNova-T2A-TagBFP2 | <https://benchling.com/s/seq-W2tTMRKypdNSlbx54WwI> |
| CAG_promoter-membrane_localization-SuperNova2-T2A-TagBFP2 | <https://benchling.com/s/seq-tq4MQ7BvjfC0mVKJDaQ4> |
| CMV_promoter-EGFP_NLS | <https://benchling.com/s/seq-tWUaqezthtthWw1LHpvU> |
| KillerRed in modified pBAD vector | <https://benchling.com/s/seq-ohaqgrbhNP2qg1aiwu9T> |
| KillerRed2 in modified pBAD vector | <https://benchling.com/s/seq-PJlxAWV5EecQeJVx2b3N> |
| SuperNova in modified pBAD vector | <https://benchling.com/s/seq-6wduGoA3pAZ0e7yDZfAf> |
| SuperNova2 in modified pBAD vector | <https://benchling.com/s/seq-6v8cMA5TakhSwNpfUwVV> |
